# Postoperative complications of ultrasound-guided inferior alveolar nerve and maxillary nerve blocks: a retrospective study

**DOI:** 10.1186/s40981-022-00533-4

**Published:** 2022-06-16

**Authors:** Yuki Kojima, Takeshi Murouchi, Naoko Okayama, Kazuma Asano, Masakazu Akiba, Junichiro Hamasaki

**Affiliations:** 1 Department of Anesthesiology, Asahi General Hospital, I-1326 Asahi-shi, Chiba, 289-2511 Japan; 2 Department of Dental Anesthesiology, Field of Oral and Maxillofacial Rehabilitation, Kagoshima University Graduate School of Medical and Dental Sciences, Kagoshima, Japan; 3 Department of Anesthesiology, Kagoshima City Hospital, Kagoshima, Japan; 4 Department of Dentistry and Maxillofacial Surgery, Asahi General Hospital, Chiba, Japan

**Keywords:** Maxillofacial surgery, Ultrasound-guided inferior alveolar nerve blocks, Ultrasound-guided maxillary nerve blocks, Local anesthetic, Complication

To the Editor,

Recent studies have reported that ultrasound-guided trigeminal nerve blocks are effective as a postoperative analgesic method in maxillofacial surgery [1–4]. These mainly include ultrasound-guided inferior alveolar nerve blocks (IANBs), also called mandibular nerve blocks, and ultrasound-guided maxillary nerve blocks (MNBs) [5]. These nerve blocks are not widely used, and there are no reports on their associated complications. Statistical analyses of the complications are important for demonstrating the safety of a technique to facilitate a prompt response to common complications. It is also necessary to provide patients with a clear explanation regarding the risks associated with the procedure that they will undergo to obtain informed patient consent. In this study, we retrospectively investigated the rate of complications in patients who underwent ultrasound-guided IANBs and MNBs at multiple institutions.

This retrospective cohort study was conducted and reported according to the STROBE checklist. All methods were performed according to the relevant guidelines and regulations. The study was conducted across three general hospitals in Japan. This study is registered in a publicly accessible database (UMIN Clinical Trials Registry ID: UMIN000045581). We collected the data of all patients who underwent ultrasound-guided IANBs and MNBs between April 1, 2018 and March 31, 2021. Eligible patients were identified from a database of clinical records (Fig. 1). Ultrasound-guided IANBs and MNBs were performed using the extraoral approach before surgery. The local anesthesia (LA) used in all cases was ropivacaine.

**Fig. 1:**
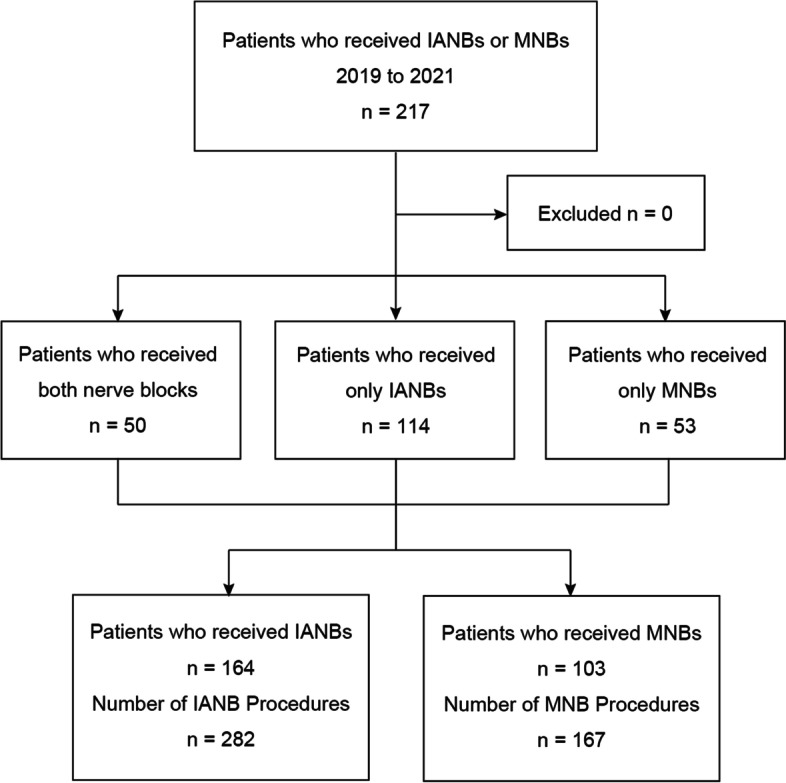
Flow diagram of the study population. *IANB* ultrasound-guided inferior alveolar nerve block, *MNB* ultrasound-guided maxillary nerve block

The following items were considered as possible complications: LA toxicity, allergies, neuropathy, movement disorders, pain in the punctured area, infection, sensory deficits, and blood vessel damage. During the study period, 217 patients underwent ultrasound-guided IANBs and MNBs (Fig. 1). The number of patients who underwent ultrasound-guided IANBs (IANB group) was 164, and the total number of procedures was 282. The number of patients who underwent ultrasound-guided MNBs (MNB group) was 103 patients, and the total number of procedures was 167 (Tables 1 and 2). No complications were observed in both groups.

**Table 1:** Patient characteristics

Characteristics	IANB group	MNB group
Number of patients	164	103
Number of procedures	282	167
Demographics		
Age, mean (years)	47 ± 21	41 ± 24
Under 10 years old, n	1	5
Weight, mean (kg)	59 ± 12	56 ± 15
Height, mean (cm)	159 ± 9	157 ± 14
BMI, mean (kg/m^2^)	23±4	22±4
Male, n (%)	67 (40)	43 (41)
ASA-PS		
I	62	50
II	86	47
III	16	6
Operation time, mean (min)	128 ± 125	136 ± 96
Anesthesia time, mean (min)	187 ± 137	209 ± 105
Inpatient, *n*	149	103
Outpatient, *n*	15	0

±SD

*IANB* inferior alveolar nerve block, *MNB* maxillary nerve block, *BMI* body mass index, *ASA-PS* American Society of Anesthesiologists’ physical status, *SD* standard deviation

**Table 2:** Nerve block characteristics

Characteristics	IANB group	MNB group
Unilateral	46	39
Bilateral	118	64
Side		
Left	140	84
Right	142	83
LA volume		
5 mL	66	71
6 mL	68	75
10 mL	110	0
Other	38	21
LA concentration		
0.2%	13	10
0.375%	269	157

*IANB* inferior alveolar nerve block, *MNB* maxillary nerve block, *LA* local anesthesia

Since the ultrasound-guided approach can be performed while confirming the anatomical findings and checking the injection range, the occurrence of the aforementioned complications may be reduced. In addition, IANBs and MNBs are peripheral nerve blocks that are categorized as compartment nerve blocks. Therefore, we considered that they have a low risk of damage to the targeted nerves. To include rare complications, it is necessary to collect and analyze more data on IANBs and MNBs.

## Abbreviations

LA: Local anesthetic
NSAIDs: Nonsteroidal anti-inflammatory drugs
IANB: Inferior alveolar nerve block
MNB: Maxillary nerve block
